# Role of Ceftazidime-Avibactam in Urinary Tract Infections Caused by Carbapenem-Resistant Enterobacterales

**DOI:** 10.7759/cureus.75221

**Published:** 2024-12-06

**Authors:** Shipra Dobhal, Manodeep Sen, Harshita Yadav, Jyotsana Agarwal, Anupam Das, Abhilash Chandra, Alok Srivastava, Soumya Nath

**Affiliations:** 1 Microbiology, Dr. Ram Manohar Lohia Institute of Medical Sciences, Lucknow, IND; 2 Nephrology, Dr. Ram Manohar Lohia Institute of Medical Sciences, Lucknow, IND; 3 Urology, Dr. Ram Manohar Lohia Institute of Medical Sciences, Lucknow, IND; 4 Anesthesia, Dr. Ram Manohar Lohia Institute of Medical Sciences, Lucknow, IND

**Keywords:** carbapenem-resistant enterobacterales (cre), ceftazidime-avibactam, new delhi metallo ß lactamases (ndm), synergy, urinary tract infection

## Abstract

Introduction

Urinary tract infections (UTIs) are one of the most common bacterial infections encountered in community and healthcare settings. Increasing antimicrobial resistance patterns worldwide have limited the treatment options available. Overuse of carbapenems which were considered as the last resort for multi-drug resistant UTIs over the past decade has led to the emergence of carbapenem-resistant Enterobacterales (CRE). Ceftazidime-avibactam is a novel beta-lactam inhibitor combination drug indicated for the treatment of complicated UTIs caused by CRE.

Materials and methods

This was a prospective, observational study conducted in the Department of Microbiology at Dr. Ram Manohar Lohia Institute of Medical Sciences, Lucknow from January 1, 2022, to June 30, 2022. A total of 1716 urine samples were processed for identification and antimicrobial susceptibility testing. This research was approved by the Institutional Ethics Committee (IEC) of Dr. Ram Manohar Lohia Institute of Medical Sciences, Lucknow, Uttar Pradesh, India, under IEC number 140/20. Isolates belonging to Enterobacterales and showing resistance to carbapenems were selected for further processing. Epidemiological details and the antimicrobial susceptibility profile of all patients with CRE uropathogens were compared. These isolates were then tested for susceptibility to ceftazidime-avibactam (30/20 μg) disc using the Kirby-Bauer disc diffusion method. A subset (n= 20) of CRE isolates were chosen for gene detection using multiplex polymerase chain reaction followed by synergy testing using ceftazidime-avibactam and aztreonam gradient strip-based susceptibility testing.

Results

In the 1716 samples processed, only 28.1% samples had significant growth out of which 15.3% isolates belonged to Enterobacterales and showed in-vitro resistance to imipenem/meropenem. CRE were approximately two times more common in men as compared to women in the age group of 41-60 years (31.1%). The majority were inpatient samples (72%). The antimicrobial susceptibility profile of the CRE uropathogens showed maximum susceptibility to fosfomycin (66.7%), nitrofurantoin (30.7%), and aminoglycosides (13.5%) and maximum resistance to norfloxacin (100%), cefazolin (98.7%), and netilmicin (98.7%). Ceftazidime-avibactam had an in vitro resistance of 91.9%. The most common gene detected was NDM followed by KPC. The isolates that were resistant to ceftazidime-avibactam and positive for NDM gene (n=20) on being tested for phenotypic synergy using ceftazidime-avibactam and aztreonam E strips showed 100% susceptibility.

Conclusion

Carbapenem-resistant gram-negative pathogens have become a major healthcare burden. They limit the treatment options available thereby increasing the morbidity and mortality in patients. Carbapenems which were at one time considered as the last line of treatment are deemed ineffective by these superbugs. CRE are among the most dreaded infectious agents. Ceftazidime-avibactam has been suggested as a therapeutic option for the management of CRE but in our study ceftazidime-avibactam alone had a high in-vitro resistance. Nonetheless, the combination of ceftazidime-avibactam and aztreonam showed good in-vitro susceptibility. Synergistic combination of these two antimicrobials can be considered as a treatment option, especially in regions where NDM is prevalent. Research is still limited regarding the clinical efficacy of this combination.

## Introduction

Urinary tract infections (UTIs) are one of the common bacterial infections encountered in community and healthcare settings [1]. A complicated UTI (cUTI) is one that occurs because of anatomical, functional, or pharmacological factors that predispose the patient to either persistent infection, recurrent infection, or treatment failure. Treatment for cUTIs may require a 10- to 14-day course of therapy [2]. Increasing antimicrobial resistance patterns worldwide have limited the treatment options available. Overuse of carbapenems which were considered the last resort for multi-drug resistant UTIs over the past decade has led to the emergence of carbapenem-resistant Enterobacterales (CRE) and the most important mechanism attributed to the development of resistance to carbapenems is the production of carbapenemase enzyme by the bacteria which inactivates the drug [3]. Ceftazidime-avibactam was approved by the Food and Drug Administration (FDA) as a treatment option in February 2015. It is a novel beta-lactam inhibitor combination drug indicated for cUTIs, complicated ventilator-associated pneumonia, and intra-abdominal infections caused by CRE [4].

The aim of our study was to test the efficacy of ceftazidime-avibactam in CRE urinary isolates from patients with UTI at a tertiary care level super specialty Institute in North India. Our objectives were to determine the prevalence of CRE urinary isolates from cases with UTI followed by assessing the susceptibility of ceftazidime-avibactam for these isolates by the Kirby-Bauer method. We also performed genotypic characterization of a subset of CRE urinary isolates and susceptibility testing for ceftazidime-avibactam plus aztreonam combination.

## Materials and methods

This was a prospective, observational study conducted in the Department of Microbiology at Dr. Ram Manohar Lohia Institute of Medical Sciences, Lucknow from January 1, 2022 to June 30, 2022. A sample size of 1716 was calculated as it produced a two-sided 95% confidence interval with a width equal to 0.010 when the sample proportion was 0.010 [5]. The Institutional Ethics Committee (IEC) of Dr. Ram Manohar Lohia Institute of Medical Sciences, Lucknow, Uttar Pradesh, India, approved this research study under IEC number 140/20. A total of 1716 urine samples were processed for uropathogen identification and antimicrobial susceptibility testing. Culture-positive urine samples of CRE with a colony count of ≥10^5^ colony-forming units/ml and ≥10^3^ colony-forming units/ml in the case of catheterized patients were considered significant and were included in the study [6]. Contaminated urine samples, urine samples with a colony count of less than 10^2^ colony forming units/ml, and unwilling patients were excluded from our study. Urinary pathogens isolated were identified up to the species level and antimicrobial susceptibility testing was done using the Kirby-Bauer disc diffusion method. Isolates belonging to Order Enterobacterales and showing resistance to Imipenem/Meropenem discs were selected as CRE for further processing. The antimicrobial susceptibility profiles of CRE isolates were compared and then they were tested for carbapenemase production using the modified carbapenem inactivation method (mCIM) [7]. All the CRE isolates were checked for susceptibility to ceftazidime-avibactam using the Kirby-Bauer disc diffusion method. A subset of isolates (n= 20) was tested for gene detection using a HiMedia Carbapenemase gene detection kit (multiplex real-time probe-based PCR) followed by synergy of the ceftazidime-avibactam Ezy MIC strip and HiMedia Aztreonam Ezy MIC strip by the gradient strip stacking method [8].

## Results

**Figure 1:**
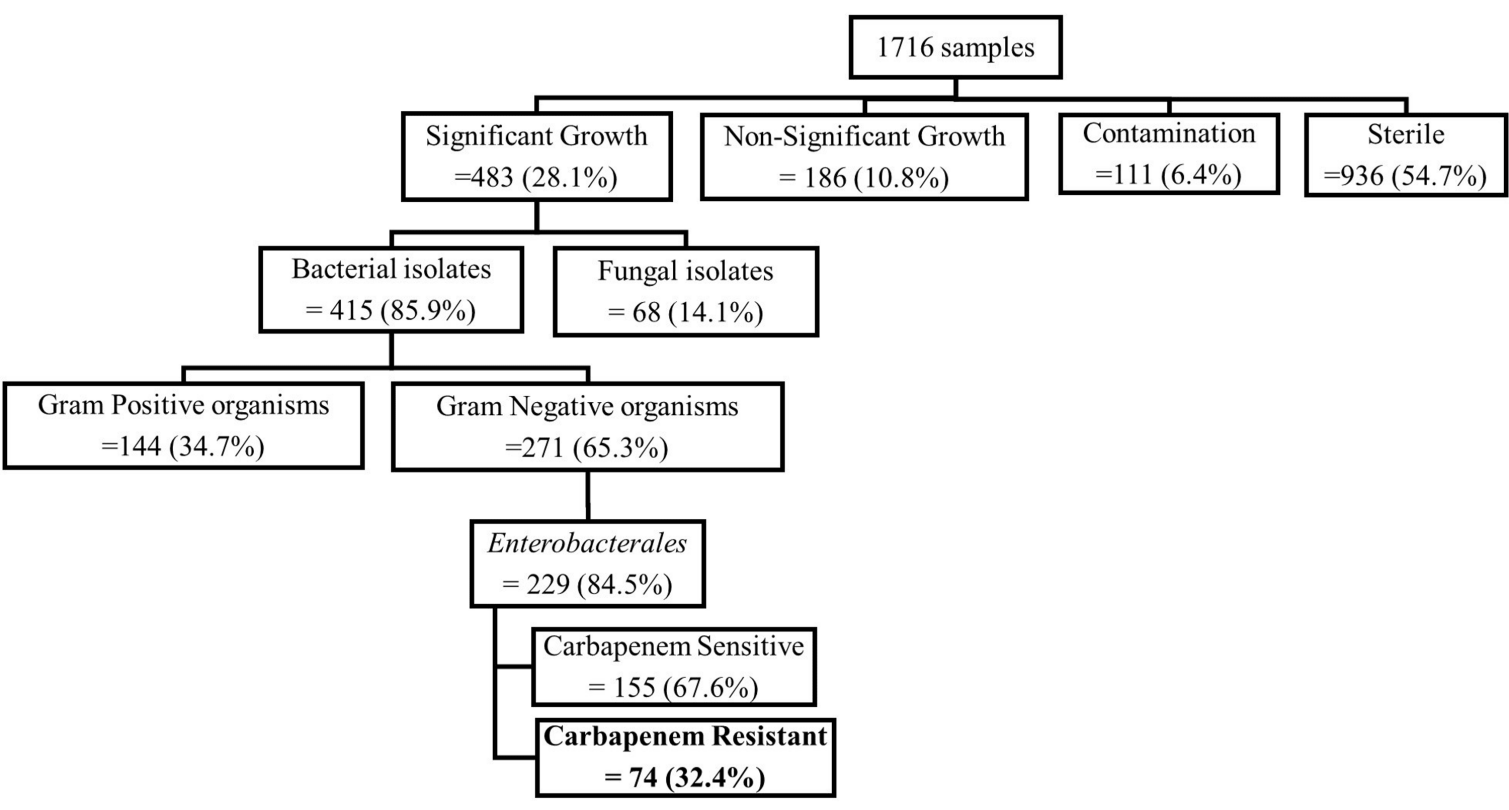
Flowchart of urine samples received and processed in our laboratory

In the 1716 samples processed, only 483 (28.1%) samples had significant growth out of which 74 (15.3%) isolates belonged to Enterobacterales and showed in-vitro resistance to imipenem/meropenem. Among these, the most common organism was *E. coli* (78%) followed by *K. pneumoniae* (22%) (Figure 1).

CRE were approximately two times more common in men as compared to women in the age group of 41-60 years (31.1%). The majority were inpatient samples (72%). In 45 out of 74 inpatient cases, 34% cases were patients admitted to wards and 62% were Intensive Care Unit (ICU) patients. The most common clinical presentation in outpatients was found to be fever followed by urgency and frequency (72.4%), burning micturition (37.9%), and pain abdomen (27.6%), whereas for inpatients, it was fever (66.6%) followed by pain in the abdomen (11.1%). Comorbidities were present in 74.6% (n=53) of patients with CRE in urine. The most common co-morbidity was diabetes mellitus. On assessing the presence of catheterization in patients, we found that 69% of cases had an indwelling catheter present.

**Figure 2:**
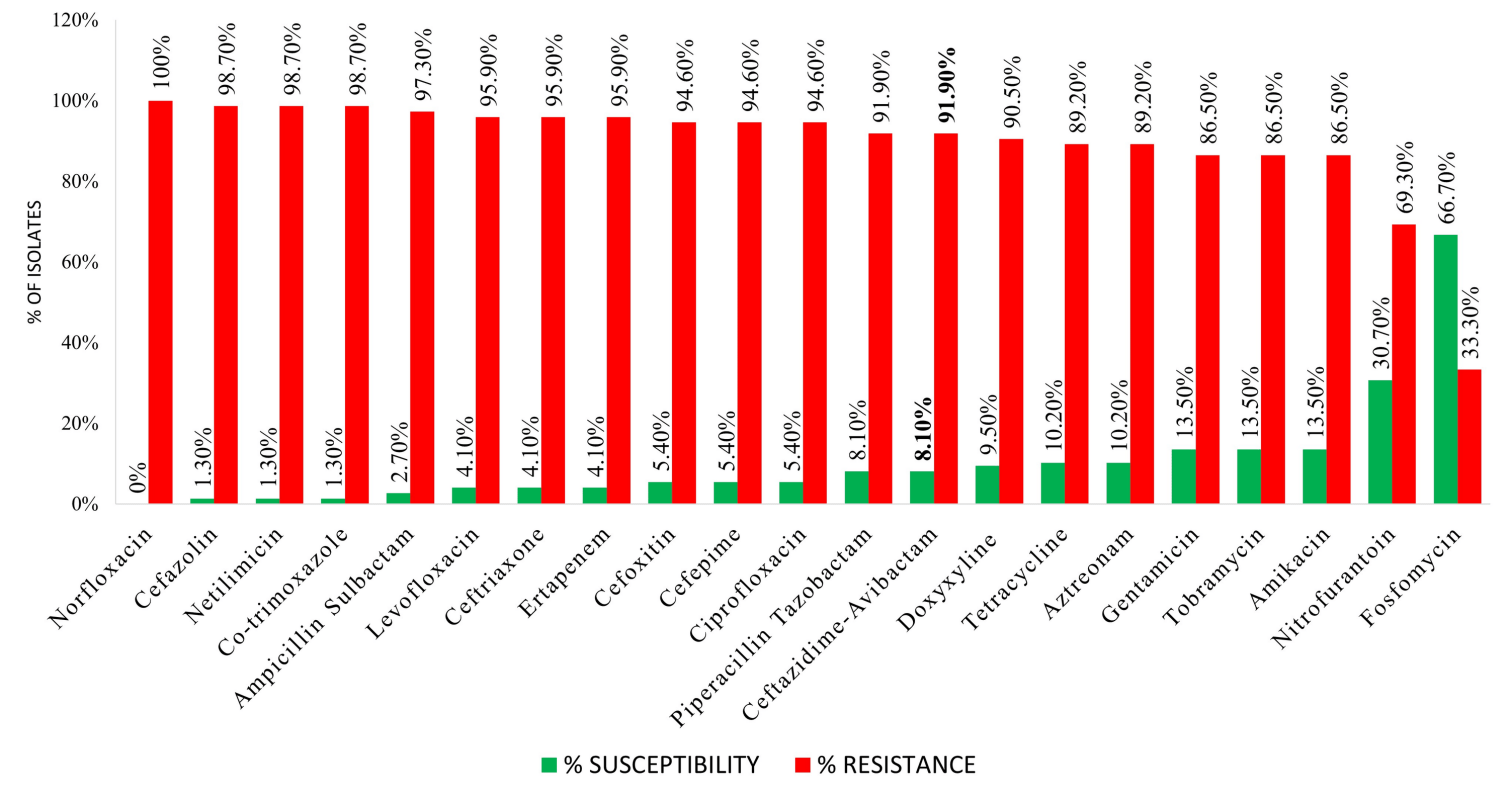
Antimicrobial susceptibility profile of all carbapenem-resistant Enterobacterales isolated from the urine (n=74)

The antimicrobial susceptibility profile of all 74 CRE uropathogens showed maximum susceptibility to fosfomycin (66.7%), nitrofurantoin (30.7%), amikacin, and tobramycin (13.5% each) and maximum resistance to norfloxacin (100%), cefazolin (98.7%), netilmicin (98.7%), co-trimoxazole (98.7%), and ampicillin-sulbactam (97.3%). Ceftazidime-avibactam on being tested had an in vitro resistance of 91.9% and susceptibility of 8.1% (Figure 2).

89.2% of CRE isolates were positive for carbapenemase production using mCIM and 86.5% of isolates were positive for metallo-beta-lactamase (MBL) production. Genotypic detection of carbapenemases by real-time multiplex PCR testing was done on a subset of isolates (n=20) for identification of the underlying carbapenemase gene. The most common gene detected was NDM in 100% (n=20) isolates followed by KPC in 75% (n=15) isolates. Among the oxacillinase genes, OXA-48 was detected in 60% (n=12) isolates and OXA-58 was detected in 15% (n=3) isolates. The most common combination detected was NDM+KPC+OXA-48 in 45% (n=9) isolates.

**Figure 3:**
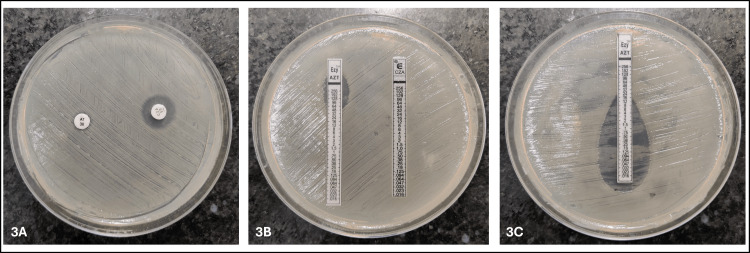
A: CRE uropathogen showing resistance to aztreonam and ceftazidime-avibactam discs; B: Same isolate showing resistance to aztreonam and ceftazidime-avibactam E-strips; C: Same isolate showing synergy on using ceftazidime-avibactam and aztreonam E-strip by the gradient strip stacking method CRE: Carbapenem-resistant enterobacterales

The isolates that were resistant to ceftazidime-avibactam and positive for NDM gene (n=20) on being tested for phenotypic synergy using ceftazidime-avibactam and aztreonam E strips showed 100% susceptibility (Figure 3).

## Discussion

Carbapenem-resistant gram-negative pathogens have become a major healthcare burden [9]. CRE are defined by Centers for Disease Control and Prevention (CDC) as an Enterobacterales isolate that is resistant to ertapenem/imipenem/meropenem/doripenem according to the current Clinical and Laboratory Standards Institute (CLSI) breakpoints or documentation of carbapenemase production by the isolate [10]. In the present study amongst the culture-positive isolates, 47.4% belonged to Enterobacterales and 15.3% (n=74) isolates were categorized as CRE.

The commonest organism in our study was *E. coli *(80%) followed by *K. pneumoniae *(20%). This was in accordance with various studies reported in India [11,12]. Like our study, various studies by Hu et al. (2012), Veeraraghavan et al. (2017), and Zhang et al. (2021) reported a susceptibility of 10-30% to Amikacin and Tetracycline in Carbapenemase-producing Enterobacterales which are usually pan-drug resistant [11-13].

CRE frequency was highest in men in the age group of 40-60 years. We evaluated the various risk factors for CRE urinary pathogens. Shields et al. (2021) reported that the majority of patients with Carbapenem-resistant organisms causing UTI had multiple underlying co-morbidities, in our study the most common co-morbidity identified was diabetes mellitus [14]. Also, patients with medical devices, undergoing invasive procedures, or being admitted to the ICU were seen to be at a particular risk for CRE [15]. Urinary catheterization also is an important risk factor in these cases [16].

Carbapenemases are enzymes that hydrolyze nearly all β-lactam antibiotics, including carbapenems. Due to their stable gene transfer mechanisms, they can defy geographical boundaries and hence become difficult to control. PCR testing of CRE isolated from urine from various studies in the Indian subcontinent has revealed NDM followed by OXA48-like most commonly in *E. coli* isolates [17,18]. In contrast to this iCREST, Spain has reported OXA-48 (86.8%) as the most common carbapenemase followed by KPC (6.9%), VIM (4.8%), NDM (1.1%), and IMP (0.6%) in Enterobacterales isolated from urine sample [19]. Co-carriage of multiple genes poses a major threat by limiting the antimicrobial activity by increasing the antimicrobial hydrolysis spectrum of the organism and their presence on plasmids increases their ability for horizontal transfer to multiple species [20]. In our study, 75% of isolates showed co-carriage of multiple carbapenemase genes but their phenotypic expression was not detected. This alerts us regarding the need for a keen observance to promote early detection of production of multiple carbapenemases by organisms which may further restrict the pipeline of antimicrobials available for these patients. Surveillance and judicious use of infection prevention and control practices should be implemented to address the rapid intercontinental spread of carbapenemase-producing organisms.

Ceftazidime is a third-generation cephalosporin and Avibactam is a novel non-β-lactam β-lactamase inhibitor, designed around a diaza-bicyclo octane structure instead of a ß-lactam core. Avibactam covalently binds to the β-lactamase active site hydroxyl group forming a carbamate linkage that is more rigid and persists without degradation hence leading to diminished availability of active enzyme for hydrolysis and thus decreased inactivation of the β-lactam antibiotic [21]. Ceftazidime/avibactam has in vitro activity against Ambler class A, class C, and some class D β-lactamase-producing bacteria, including Enterobacterales and Pseudomonas aeruginosa with no activity against metallo-β-lactamase producing bacteria. As per the Infectious Diseases Society of America (IDSA) and Indian Council of Medical Research (ICMR) recommendations, ceftazidime-avibactam can be used as a first-line treatment against OXA-48-like and KPC-producing CRE [4]. The INFORM Global Surveillance Study reported that the overall resistance rate of ceftazidime-avibactam in various isolates was as low as 0.5% but for MBL-positive isolates; it was 96.6% [22]. Ceftazidime-avibactam is an effective agent in vitro against meropenem-nonsusceptible Enterobacterales, except for isolates in which carbapenem resistance is mediated through MBLs [22]. In our institute on testing ceftazidime-avibactam in CRE, a high resistance rate was detected. This could be due to the high incidence of MBL enzyme production as a mechanism of resistance in our study. Out of the 74 CRE isolated from urine, 89.2% were positive for MBL detection. Ceftazidime-avibactam has good efficacy against CRE causing complicated UTIs, complicated intra-abdominal infections, and ventilator-associated pneumonia, but the downfall was its inefficacy against MBL-producing bacteria [23]. MBL is the commonest mechanism of carbapenem resistance with high NDM rates in the Indian subcontinent, hence hindering the use of this drug [24].

The solution proposed to this is the combination of ceftazidime-avibactam with aztreonam, a monobactam with good activity against MBL-producing bacteria. We performed synergy testing on a subset (n= 20) of CRE isolates with NDM gene and showed resistance to ceftazidime-avibactam. A 100% susceptibility was observed when using ceftazidime-avibactam with aztreonam, suggesting that this combination might be useful in the context of the Indian subcontinent. Ceftazidime-avibactam with aztreonam has been reported to have good antimicrobial activity against approximately 80% MBL Enterobacterales and isolates showing co-production of KPCs and ESBLs with only 3% isolates showing non-synergistic interactions or low antimicrobial efficacy [25]. Another study reported that 84% of VIM-type-producing Enterobacterales isolates showed a synergistic effect (84%) and 3 showed indifference (16%) [26]. Falcone et al. (2021) compared the clinical outcomes of patients and reported that the combination of ceftazidime-avibactam and aztreonam had lower 30-day mortality, lower clinical failure at day 14, and shorter length of stay as compared to other active antibiotics [27]. In-vitro data from various studies have shown good therapeutic potential, especially for infections caused by MBL producers, but there are very limited data on the clinical efficacy of this synergistic combination.

Limitations

Limitations of the study include being conducted in a single, tertiary care hospital setting, which might not represent the scenario of community-acquired infections; genotypic detection and synergy testing could be performed only on a subset of isolates; hence a better overview of the susceptibility pattern could not be assessed.

## Conclusions

Antimicrobial resistance is one of the major threats to public health. The frequency of CRE causing UTIs in our study was found to be 32.31%, with *E. coli f*ollowed by *Klebsiella pneumoniae* being the most common pathogens identified. Carbapenemase-producing Enterobacterales are usually pan-drug resistant, but several isolates remained susceptible to ciprofloxacin (13.5%) and amikacin (5.4%). NDM is one of the most common genotypes in Southeast Asian countries; this was supported by our study wherein NDM followed by KPC were the most common genes. A high rate of co-carriage of genes was also detected. The most common is NDM+KPC+OXA-48. Carbapenemase-producing bacteria disseminate more readily and have important treatment implications. Their monitoring is essential for better management of cases.

On testing for in-vitro susceptibility of ceftazidime-avibactam in CRE uropathogens, a high resistance rate was observed. This could be due to the high incidence of MBL enzyme production as a mechanism of resistance in our study. Out of the 74 CRE isolated from urine, 89.2% were positive for MBL detection. Ceftazidime-avibactam is an effective agent in vitro against meropenem non-susceptible Enterobacterales, except for isolates in which carbapenem resistance is mediated through MBLs. The combination of ceftazidime-avibactam with aztreonam can be considered a favorable therapeutic option for MBL-producing Enterobacterales.
